# Multimodal imaging features of pelvic ectopic liver combined with type I congenital extrahepatic portosystemic shunt (Abernethy malformation) in a child: a case report

**DOI:** 10.3389/fmed.2026.1911471

**Published:** 2026-09-28

**Authors:** Xiaodong Zhu, Huihui Lin, Xu Li, Jun Hu, Zhongwei Qiao

**Affiliations:** 1 Department of Radiology, Anhui Provincial Children's Hospital, Hefei, China; 2 Department of Radiology, Children's Hospital of Fudan University, Shanghai, China

**Keywords:** Abernethy malformation, congenital portosystemic shunt, digital subtraction angiography, ectopic liver, pediatric radiology

## Abstract

**Objective:**

To investigate the multimodal imaging features, vascular anatomical characteristics, and clinical management strategies of pelvic ectopic liver combined with Type I congenital extrahepatic portosystemic shunt (Abernethy malformation) in a child, thereby enhancing the diagnostic and therapeutic understanding of this rare complex congenital anomaly.

**Methods:**

A retrospective analysis was conducted on the complete clinical and imaging data of a 12-year-old male pediatric patient. Multimodal imaging findings from ultrasound, magnetic resonance imaging (MRI), computed tomography angiography (CTA), and digital subtraction angiography (DSA) were integrated to systematically analyze the imaging manifestations of the lesion, the blood supply characteristics of the ectopic liver and portosystemic venous malformation, and the features of vascular anatomy.

**Results:**

The patient presented with a chief complaint of dysuria and urinary dribbling for one month. Multimodal imaging revealed an irregular soft tissue mass in the pelvic cavity, connected to the left hepatic lobe via a soft tissue pedicle. The enhancement pattern on contrast-enhanced imaging was identical to that of the orthotopic liver, consistent with the imaging features of pelvic ectopic liver. CTA three-dimensional vascular reconstruction and DSA demonstrated hypoplasia of the proper hepatic artery, with its left branch abnormally coursing inferiorly to supply the pelvic ectopic liver. The superior mesenteric vein drained directly into the inferior vena cava, with no opacification of the intrahepatic portal venous system, consistent with the imaging diagnosis of type I Abernethy malformation. To our knowledge, no similar case of pelvic ectopic liver combined with type I congenital extrahepatic portosystemic shunt has been reported globally. Following multidisciplinary consultation, a conservative management strategy of long-term imaging follow-up was adopted for the patient, with no surgical intervention planned at present, and liver transplantation reserved as a definitive treatment option in the event of severe long-term complications.

**Conclusion:**

Pelvic ectopic liver combined with type I congenital extrahepatic portosystemic shunt represents an extremely rare congenital composite hepatic vascular and visceral malformation in clinical practice. The multimodal imaging system integrating ultrasound, MRI, CTA, and DSA can accurately and comprehensively delineate the location, morphology, blood supply origin of the ectopic liver, and the anatomical details of the portosystemic venous malformation. It serves as a reliable imaging basis for the diagnosis, anatomical assessment, and long-term follow-up of this rare pediatric condition, and provides critical support for the formulation of individualized clinical treatment strategies.

## Clinical presentation

The patient was a 12-year-old male admitted with “dysuria and urinary dribbling for one month.” One week prior to admission, the patient developed persistent abdominal discomfort, and the urinary symptoms remained unrelieved. Abdominal ultrasound at an external hospital suggested a huge space-occupying lesion in the abdominal cavity. The patient was referred to our hospital for further diagnosis and treatment. During the disease course, there were no accompanying symptoms such as fever, vomiting, or diarrhea.

Laboratory tests indicated mildly abnormal liver function parameters: alanine aminotransferase (ALT) 44.80 U/L, aspartate aminotransferase (AST) 46.20 U/L, and gamma-glutamyl transferase (GGT) elevated to 67.10 U/L. Total bilirubin was 15.10 μmol/L, within the normal range; direct bilirubin was 6.40 μmol/L, and total bile acids were abnormally elevated at 52.50 μmol/L. Total protein was 68.20 g/L, albumin was 42.50 g/L, with protein parameters essentially normal. Serum alpha-fetoprotein (AFP) test results were normal.

## Imaging findings

### Ultrasonography

Ultrasound examination detected a hypoechoic lesion in the region from the mid-upper abdomen to the pelvic area above the bladder, with blood flow signals visible within the lesion, extending from the mid-upper abdomen to the upper pelvis (Figure 1A).

**Figure 1:**
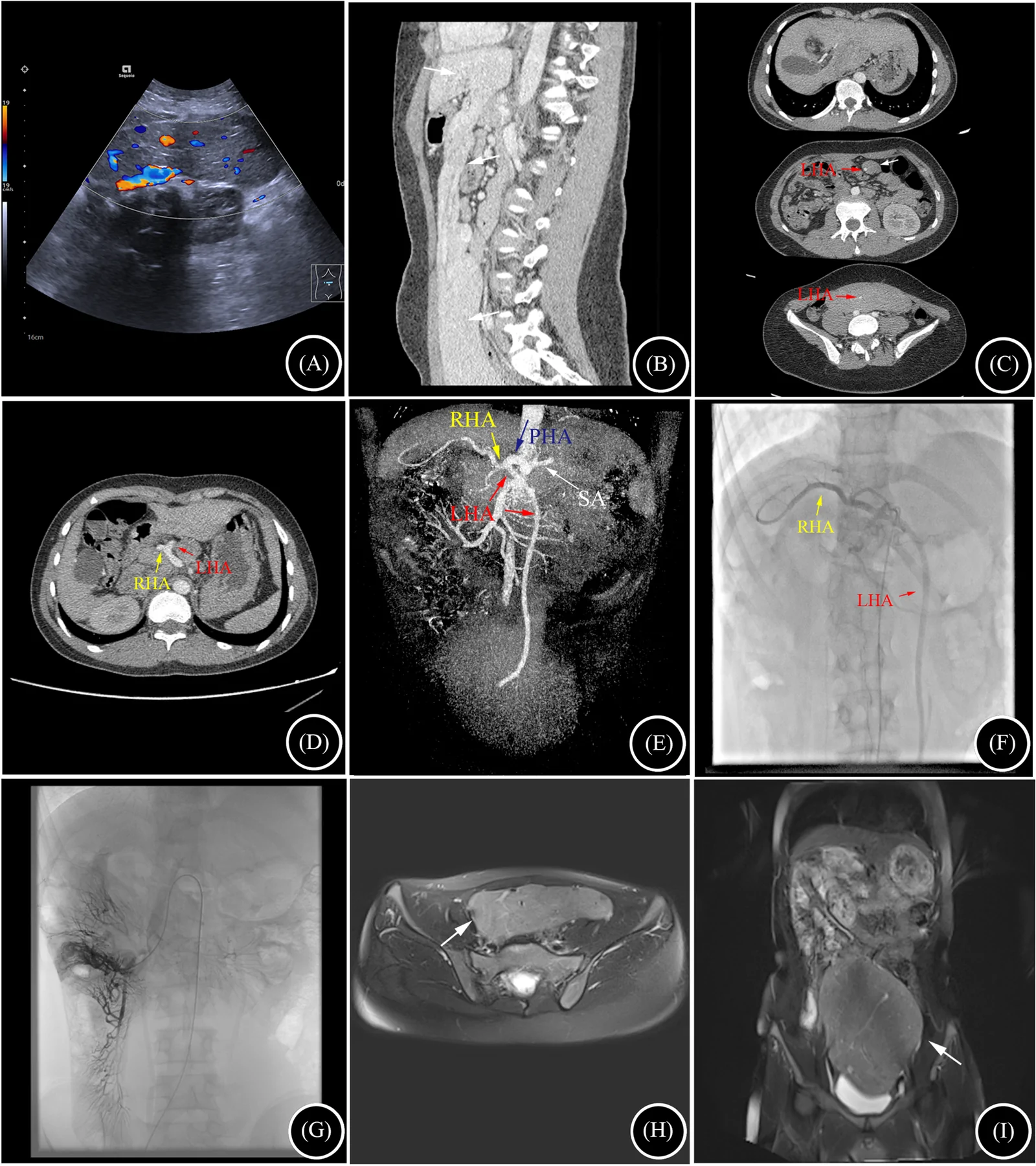
**(A)** ultrasound image showing the morphology and internal blood flow signals of the ectopic liver in the pelvic cavity. **(B)** Sagittal contrast-enhanced CT image. The upper arrow indicates the orthotopic liver, the middle arrow indicates the band-like soft tissue pedicle connecting the liver to the pelvic lesion, and the lower arrow indicates the pelvic ectopic liver. **(C)** Multi-planar axial contrast-enhanced CT image, showing the morphology and enhancement characteristics of the orthotopic liver, the soft tissue pedicle (white arrow), and the ectopic liver in the pelvic cavity; the left branch of the proper hepatic artery (red arrow) passes through the soft tissue pedicle and enters the pelvic ectopic mass to supply blood. **(D)** CT image at the bifurcation level of the proper hepatic artery, showing a partial defect in the orthotopic liver; at this level, the proper hepatic artery divides into the right branch of the proper hepatic artery (yellow arrow) and the left branch of the proper hepatic artery (red arrow). **(E,F)** CTA three-dimensional vascular reconstruction and DSA images, showing the proper hepatic artery (blue arrow) giving rise to the right branch of the proper hepatic artery (yellow arrow) and the left branch of the proper hepatic artery (red arrow); the left branch of the proper hepatic artery descends to supply the pelvic mass; the splenic artery is visible on the left side of the proper hepatic artery (white arrow). **(G)** Superior mesenteric artery angiography, showing the superior mesenteric vein directly draining into the inferior vena cava, with no normal portal venous return pathway. **(H,I)** Axial and coronal MRI images, showing the ectopic liver in the pelvic cavity (white arrow); the lesion is fusiform in shape, with slender ends and a widened middle portion, presenting typical morphological features.

### Computed tomography (CT) angiography

The morphology of the orthotopic liver in the upper abdomen of the child was markedly irregular. At the level above the gallbladder, the liver tissue morphology was intact, while at the level of the gallbladder and below, partial absence of liver tissue was observed (Figure 1D). At the bifurcation level of the proper hepatic artery, the left lobe of the liver was connected to an irregular pelvic mass in the prevertebral region via a band-like soft tissue pedicle. Enhanced scanning showed that the enhancement pattern and degree of the pelvic mass were essentially consistent with those of the orthotopic liver (Figures 1B, C). CTA three-dimensional vascular reconstruction revealed that the left branch of the proper hepatic artery coursed downward along the soft tissue pedicle, ultimately supplying the pelvic mass tissue (Figure 1E).

### Digital subtraction angiography (DSA)

Celiac trunk angiography showed that the celiac trunk gave rise to the proper hepatic artery, with its left branch extending all the way into the pelvic mass (Figure 1F). The proper hepatic artery was hypoplastic. Superior mesenteric artery angiography demonstrated direct drainage of the superior mesenteric vein into the inferior vena cava (Figure 1G). Neither superior mesenteric artery angiography nor wedged hepatic venography could visualize the portal vein.

### Magnetic resonance imaging (MRI)

MRI shows that the child's orthotopic liver in the upper abdomen has an irregular shape, and an irregular soft tissue mass with clear borders is visible anterior to the sacrum (Figures 1H, 1I).

## Discussion

Ectopic liver is a rare congenital organ developmental malformation, with the literature reporting that its most common sites are the gallbladder wall and the thoracic cavity (1). The vast majority of patients with ectopic liver present no specific clinical symptoms and are usually discovered incidentally during autopsy, abdominal surgery, or imaging examinations for other diseases (2). Previously reported sites of ectopic liver distribution include the spleen, umbilical cord, inferior vena cava, heart, and lung parenchyma (2–4). In this case, the ectopic liver was located in the pelvic cavity, adjacent to the anterior aspect of the sacrum, and connected to the orthotopic liver via a band-like soft tissue pedicle originating from the left hepatic lobe. Multi-slice CT imaging revealed a focal tissue defect in the orthotopic liver of the child, suggesting on imaging that the orthotopic liver may be underdeveloped compared with healthy children of the same age. However, precise liver volume measurement was not performed in this study, and the clinical significance of this morphological abnormality remains to be further validated. In this case, the ectopic liver was positioned unusually low, causing compression on the bladder and adjacent urinary structures, and the child's symptoms of dysuria and urinary dribbling may be secondary to this compressive effect. CTA three-dimensional vascular reconstruction and DSA celiac trunk angiography both demonstrated that the ectopic liver was supplied by the left branch of the proper hepatic artery through the soft tissue pedicle, a blood supply pattern essentially consistent with that reported by Cetin Murat et al. (5) in an adult case of upper abdominal ectopic liver.

Sumida et al. (6) previously reported an adult case of ectopic liver located anterior to the inferior vena cava, in which autopsy revealed hypoplasia or even complete absence of the portal vein within the ectopic liver. Based on this finding, the authors proposed the hypothesis that ectopic liver and congenital portal venous system malformations share a common pathogenetic mechanism. In the present case, the pelvic ectopic liver was combined with type I extrahepatic portosystemic shunt malformation of the orthotopic liver, which is highly consistent with this hypothesis and further corroborates the association between the two types of malformations.

Congenital extrahepatic portosystemic shunt (CEPS), also known as Abernethy malformation, is a rare congenital venous developmental anomaly characterized by an abnormal communication between the portal venous system and the systemic venous system, allowing mesenteric and splenic venous blood to bypass the liver and drain directly into the systemic circulation. Based on the developmental status of the intrahepatic portal vein, this condition is classified into two types: type I is characterized by complete absence of the portal vein, with all portal venous return shunted into the systemic circulation through the anomalous channel; type II is mainly manifested by portal vein hypoplasia, in which intrahepatic portal vein branches are present and the liver receives partial portal perfusion, while an extrahepatic portosystemic shunt channel coexists (6). In this case, multi-angle DSA angiography showed complete non-opacification of the portal venous system, meeting the typical imaging diagnostic criteria for type I Abernethy malformation.

The clinical manifestations of congenital extrahepatic portosystemic shunt vary greatly among individuals. Mild cases may remain asymptomatic throughout life and be discovered incidentally on imaging, whereas severe cases may present with serious complications such as hepatic encephalopathy, hepatopulmonary syndrome, and pulmonary arterial hypertension (7). To the best of our knowledge, the present case involves a combination of pelvic ectopic liver and type I Abernethy malformation, and a search of the domestic and international literature has yielded no previously reported cases of this combined malformation in either children or adults, making it an extremely rare entity. Currently, there is no unified or standardized diagnostic and therapeutic protocol for this condition. Following multidisciplinary comprehensive consultation and evaluation, the child in this case was deemed to have no immediate indication for surgical intervention. After completing screening examinations including blood ammonia, echocardiography, and peripheral oxygen saturation, no evidence of complications such as hepatic encephalopathy, hepatopulmonary syndrome, or pulmonary arterial hypertension was found. The team therefore adopted a conservative follow-up strategy for the child and formulated a long-term imaging and clinical surveillance plan. Meanwhile, long-term surgical indications were clearly defined: if the child develops new-onset hepatic encephalopathy, progressive pulmonary arterial hypertension, hepatopulmonary syndrome, persistent neurological impairment, suspected malignant transformation of liver tissue, or refractory urinary symptoms due to compression by the pelvic ectopic liver during follow-up, prompt hospital readmission for reassessment is required, and liver transplantation should be considered when necessary.

## Data Availability

The original contributions presented in the study are included in the article/Supplementary Material, further inquiries can be directed to the corresponding author.

## References

[1] Li JL Wang MH Chen W. A case of intra-adrenal ectopic liver. J Clin Exp Pathol. (2021) 37(8):1019–20. 10.13315/j.cnki.cjcep.2021.08.033

[2] Akbulut S Demyati K Ciftci F Koc C Tuncer A Sahin E. Ectopic liver tissue (choristoma) on the gallbladder: a comprehensive literature review. World J Gastrointest Surg. (2020) 12(12):534–48. 10.4240/wjgs.v12.i12.534 33437404 PMC7769742

[3] Xie CB Wu Y Huang W He TK Shi RS Zhao KF. One case of retroperitoneal ectopic hepatocellular carcinoma invading inferior vena cava with pulmonary artery embolism. J Pract Radiol. (2024) 40(2):339–40. 10.3969/j.issn.1002-1671.2024.02.041

[4] Li ZL Zhao Z Zhang TT Li XR Lian SD. One case of 1⁸F–FDG PET/CT imaging of ectopic liver in the atrial region. Chin J Nucl Med Mol Imaging. (2024) 44(4):243. 10.3760/cma.j.cn321828-20230406-00094

[5] Altay CM Hacifazlioglu C Topcuoglu OM Kul M Uysal Ramadan S. Misdiagnosed epigastric mass: ectopic liver. J Magn Reson Imaging. (2017) 45(3):761–3. 10.1002/jmri.25449 27564374

[6] Sumida S Satake N Tsuneyama K. Two ectopic liver lobes discovered incidentally at an autopsy: a case report. Cureus. (2024) 16(1):e52270. 10.7759/cureus.52270 38352103 PMC10864056

[7] Tang H Song P Wang Z Han B Meng X Pan Y. A basic understanding of congenital extrahepatic portosystemic shunt: incidence, mechanism, complications, diagnosis, and treatment. Intractable Rare Dis Res. (2020) 9:64–70. 10.5582/irdr.2020.03005 32494552 PMC7263991

